# 4-(2-Sulfanyl­idene-1,3-benzothia­zol-3-yl)butan-2-one

**DOI:** 10.1107/S1600536810048051

**Published:** 2010-11-27

**Authors:** Chao-Jun Du

**Affiliations:** a School of Biology and Chemical Engineering, Nanyang Institute of Technology, Nanyang 473004, People’s Republic of China

## Abstract

In the title compound, C_11_H_11_NOS_2_, the benzine ring is coplanar with the thia­zole ring, making a dihedral angle of 0.81 (1)°. In the crystal, adjacent mol­ecules are connected into a helical chain along the *b* axis by S⋯S contacts [3.4345 (18) Å]. These helical chains are further assembled into a three-dimensional supermolecular network by inter­molecular C—H⋯O hydrogen bond between aromatic ring H atoms and carbonyl groups.

## Related literature

For a description of the Cambridge Structural Database, see: Allen (2002 ▶). For a related structure, see: Zhu *et al.* (2009 ▶). For S⋯S contacts, see: Dai *et al.* (1997 ▶).
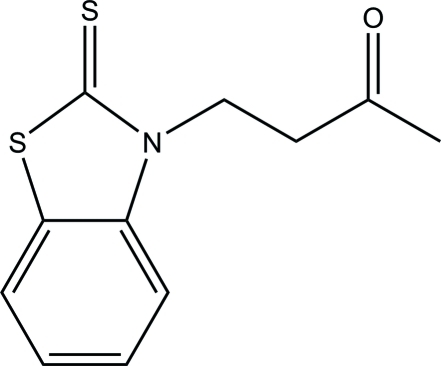


## Experimental

### 

#### Crystal data

C_11_H_11_NOS_2_
*M* *_r_* = 237.33
Orthorhombic, 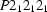

*a* = 4.9457 (19) Å
*b* = 11.586 (4) Å
*c* = 19.830 (7) Å
*V* = 1136.3 (7) Å^3^
*Z* = 4
Mo *K*α radiation
μ = 0.44 mm^−1^
*T* = 296 K
0.23 × 0.19 × 0.15 mm

#### Data collection

Bruker APEXII CCD diffractometer
Absorption correction: multi-scan (*SADABS*; Bruker, 2006 ▶) *T*_min_ = 0.905, *T*_max_ = 0.936
12565 measured reflections
1476 independent reflections
1176 reflections with *I* > 2σ(*I*)
*R*_int_ = 0.036

#### Refinement

*R*[*F* ^2^ > 2σ(*F* ^2^)] = 0.043
*wR*(*F* ^2^) = 0.117
*S* = 1.07
1476 reflections
120 parameters
2 restraints
H-atom parameters constrained
Δρ_max_ = 0.27 e Å^−3^
Δρ_min_ = −0.31 e Å^−3^

### 

Data collection: *APEX2* (Bruker, 2006 ▶); cell refinement: *SAINT* (Bruker, 2006 ▶); data reduction: *SAINT*; program(s) used to solve structure: *SHELXS97* (Sheldrick, 2008 ▶); program(s) used to refine structure: *SHELXL97* (Sheldrick, 2008 ▶); molecular graphics: *SHELXTL* (Sheldrick, 2008 ▶); software used to prepare material for publication: *SHELXTL*.

## Supplementary Material

Crystal structure: contains datablocks I, global. DOI: 10.1107/S1600536810048051/ds2068sup1.cif

Structure factors: contains datablocks I. DOI: 10.1107/S1600536810048051/ds2068Isup2.hkl

Additional supplementary materials:  crystallographic information; 3D view; checkCIF report

## Figures and Tables

**Table 1:** Hydrogen-bond geometry (Å, °)

*D*—H⋯*A*	*D*—H	H⋯*A*	*D*⋯*A*	*D*—H⋯*A*
C11—H11*B*⋯O1^i^	0.96	2.60	3.541 (5)	167

Symmetry code: (i) 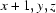.

**Fig. 1.**
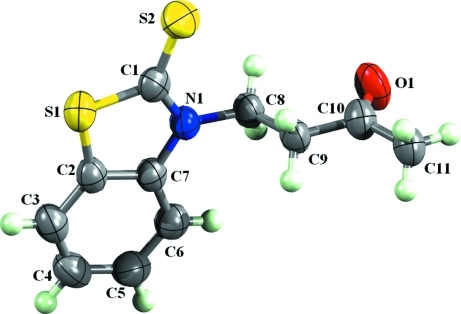
The structure of the title compound with 50% probability displacement ellipsoids.

**Fig. 2.**
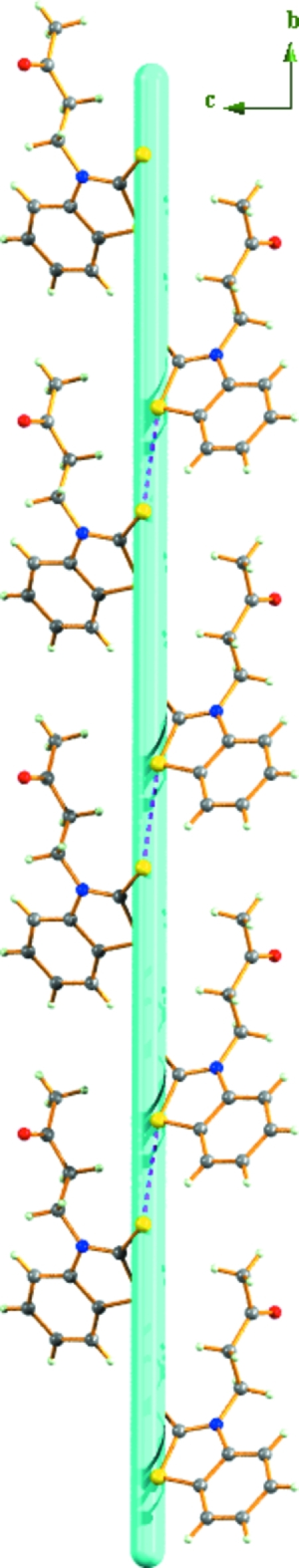
View of right-handle helical chain connected by S···S contacts along b axis.

**Fig. 3.**
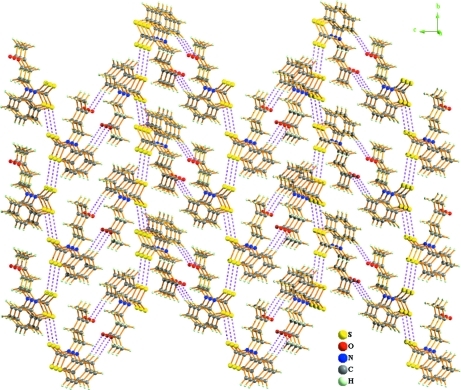
View of three-dimensional supermolecular network connected by S···S contacts and C—H···O hydrogen bonds along the c direction.

## supplementary crystallographic information

### Experimental

A solution of benzothiazole-2-thiol (167.2 mg, 1.00 mmol) and in acetone (15 ml) was slowly added to a solution of CH_2_Cl_2 _(170.0 mg, 2.00 mmol) in acetone (15 ml). The resultant solution was stirred and refluxed for 16 h and then filtered. Colorless crystals suitable for X-ray diffraction were obtained in about two weeks by slow diffusion of diethyl ether into a dilute solution of the title compound in methanol. yield: *ca* 35.8% (based on benzothiazole-2-thiol).

### Refinement

The structure was solved using direct methods followed by Fourier synthesis. Non-H atoms were refined anisotropically. All of H atoms were placed in idealized positions (C—H = 0.93, 0.96 or 0.97 Å), forced to ride on the atom to which they are bonded, and were included in the refinement in the riding-model approximation. *U*_iso_ values were set equal to 1.5*U*_eq_(parent atom) for methylic H atom and to 1.2*U*_eq_(parent atom)for all other H atoms. Friedel opposites were merged as the data could not resolve the absolute structure and consequently, the Flack paarameter was not reported.

### Crystal data

**Table:**

C_11_H_11_NOS_2_	*F*(000) = 496
*M**_r_* = 237.33	*D*_x_ = 1.387 Mg m^−^^3^
Orthorhombic, *P*2_1_2_1_2_1_	Mo *K*α radiation, λ = 0.71073 Å
Hall symbol: P 2ac 2ab	Cell parameters from 2911 reflections
*a* = 4.9457 (19) Å	θ = 2.7–22.7°
*b* = 11.586 (4) Å	µ = 0.44 mm^−^^1^
*c* = 19.830 (7) Å	*T* = 296 K
*V* = 1136.3 (7) Å^3^	Block, colorless
*Z* = 4	0.23 × 0.19 × 0.15 mm

### Data collection

**Table:**

Bruker APEXII CCD diffractometer	1476 independent reflections
Radiation source: fine-focus sealed tube	1176 reflections with *I* > 2σ(*I*)
graphite	*R*_int_ = 0.036
φ and ω scans	θ_max_ = 27.0°, θ_min_ = 2.0°
Absorption correction: multi-scan (*SADABS*; Bruker, 2006)	*h* = −6→6
*T*_min_ = 0.905, *T*_max_ = 0.936	*k* = −14→14
12565 measured reflections	*l* = −25→25

### Refinement

**Table:**

Refinement on *F*^2^	Primary atom site location: structure-invariant direct methods
Least-squares matrix: full	Secondary atom site location: difference Fourier map
*R*[*F*^2^ > 2σ(*F*^2^)] = 0.043	Hydrogen site location: inferred from neighbouring sites
*wR*(*F*^2^) = 0.117	H-atom parameters constrained
*S* = 1.07	*w* = 1/[σ^2^(*F*_o_^2^) + (0.0535*P*)^2^ + 0.413*P*] where *P* = (*F*_o_^2^ + 2*F*_c_^2^)/3
1476 reflections	(Δ/σ)_max_ < 0.001
120 parameters	Δρ_max_ = 0.27 e Å^−^^3^
2 restraints	Δρ_min_ = −0.31 e Å^−^^3^

### Special details

**Table:**

Geometry. All e.s.d.'s (except the e.s.d. in the dihedral angle between two l.s. planes) are estimated using the full covariance matrix. The cell e.s.d.'s are taken into account individually in the estimation of e.s.d.'s in distances, angles and torsion angles; correlations between e.s.d.'s in cell parameters are only used when they are defined by crystal symmetry. An approximate (isotropic) treatment of cell e.s.d.'s is used for estimating e.s.d.'s involving l.s. planes.
Refinement. Refinement of *F*^2^ against ALL reflections. The weighted *R*-factor *wR* and goodness of fit *S* are based on *F*^2^, conventional *R*-factors *R* are based on *F*, with *F* set to zero for negative *F*^2^. The threshold expression of *F*^2^ > σ(*F*^2^) is used only for calculating *R*-factors(gt) *etc*. and is not relevant to the choice of reflections for refinement. *R*-factors based on *F*^2^ are statistically about twice as large as those based on *F*, and *R*- factors based on ALL data will be even larger.

### Fractional atomic coordinates and isotropic or equivalent isotropic displacement parameters (Å^2^)

**Table:**

	*x*	*y*	*z*	*U*_iso_*/*U*_eq_	
C2	0.4190 (4)	−0.16020 (16)	0.16706 (8)	0.0559 (8)	
C3	0.5987 (5)	−0.25022 (15)	0.15525 (10)	0.0688 (10)	
H3	0.6161	−0.3094	0.1866	0.083*	
C4	0.7524 (5)	−0.25173 (19)	0.09657 (12)	0.0762 (11)	
H4	0.8726	−0.3120	0.0887	0.091*	
C5	0.7264 (5)	−0.1632 (2)	0.04970 (10)	0.0727 (10)	
H5	0.8293	−0.1642	0.0104	0.087*	
C6	0.5468 (5)	−0.07320 (17)	0.06150 (9)	0.0635 (9)	
H6	0.5294	−0.0140	0.0301	0.076*	
C7	0.3931 (4)	−0.07169 (15)	0.12018 (10)	0.0517 (8)	
S1	0.2105 (2)	−0.13581 (7)	0.23522 (4)	0.0652 (2)	
S2	−0.1380 (2)	0.07257 (8)	0.23950 (6)	0.0796 (3)	
O1	0.0395 (7)	0.3256 (2)	0.04435 (16)	0.0938 (9)	
N1	0.2077 (6)	0.0109 (2)	0.13995 (13)	0.0527 (6)	
C1	0.0920 (7)	−0.0080 (3)	0.20141 (17)	0.0563 (8)	
C8	0.1402 (8)	0.1127 (2)	0.09994 (17)	0.0595 (9)	
H8A	−0.0493	0.1312	0.1064	0.071*	
H8B	0.1670	0.0953	0.0526	0.071*	
C9	0.3097 (8)	0.2163 (3)	0.11870 (18)	0.0604 (9)	
H9A	0.2941	0.2295	0.1668	0.072*	
H9B	0.4978	0.1995	0.1089	0.072*	
C10	0.2300 (5)	0.3236 (2)	0.08225 (17)	0.0677 (7)	
C11	0.3976 (7)	0.4283 (2)	0.09581 (18)	0.0677 (7)	
H11A	0.3297	0.4919	0.0699	0.102*	
H11B	0.5818	0.4132	0.0833	0.102*	
H11C	0.3892	0.4469	0.1429	0.102*	

### Atomic displacement parameters (Å^2^)

**Table:**

	*U*^11^	*U*^22^	*U*^33^	*U*^12^	*U*^13^	*U*^23^
C2	0.0587 (19)	0.0440 (15)	0.0651 (18)	−0.0037 (15)	−0.0112 (17)	−0.0018 (14)
C3	0.071 (2)	0.0540 (17)	0.081 (2)	0.0062 (19)	−0.015 (2)	0.0014 (18)
C4	0.066 (2)	0.0627 (19)	0.100 (3)	0.010 (2)	−0.004 (2)	−0.011 (2)
C5	0.068 (2)	0.069 (2)	0.081 (2)	−0.005 (2)	0.008 (2)	−0.0089 (19)
C6	0.064 (2)	0.0609 (18)	0.066 (2)	−0.0031 (19)	−0.0016 (18)	0.0002 (17)
C7	0.0539 (19)	0.0446 (14)	0.0566 (16)	−0.0047 (15)	−0.0070 (16)	−0.0022 (13)
S1	0.0826 (6)	0.0508 (4)	0.0623 (4)	−0.0036 (5)	−0.0010 (5)	0.0044 (4)
S2	0.0827 (7)	0.0613 (5)	0.0950 (6)	−0.0010 (5)	0.0162 (6)	−0.0161 (5)
O1	0.0917 (17)	0.0742 (15)	0.115 (2)	0.0104 (17)	−0.0352 (14)	0.0259 (16)
N1	0.0536 (15)	0.0448 (12)	0.0598 (14)	−0.0007 (13)	−0.0075 (14)	0.0015 (11)
C1	0.0573 (19)	0.0456 (15)	0.0659 (18)	−0.0085 (16)	−0.0055 (18)	−0.0084 (14)
C8	0.058 (2)	0.0501 (15)	0.0710 (19)	0.0005 (16)	−0.0130 (17)	0.0067 (15)
C9	0.055 (2)	0.0501 (16)	0.076 (2)	0.0044 (17)	−0.0083 (19)	0.0091 (15)
C10	0.0791 (17)	0.0570 (12)	0.0671 (14)	0.0039 (15)	−0.0077 (12)	0.0097 (12)
C11	0.0791 (17)	0.0570 (12)	0.0671 (14)	0.0039 (15)	−0.0077 (12)	0.0097 (12)

### Geometric parameters (Å, °)

**Table:**

C2—C3	1.3900	O1—C10	1.206 (4)
C2—C7	1.3900	N1—C1	1.364 (4)
C2—S1	1.724 (2)	N1—C8	1.460 (4)
C3—C4	1.3900	C8—C9	1.511 (4)
C3—H3	0.9300	C8—H8A	0.9700
C4—C5	1.3900	C8—H8B	0.9700
C4—H4	0.9300	C9—C10	1.491 (4)
C5—C6	1.3900	C9—H9A	0.9700
C5—H5	0.9300	C9—H9B	0.9700
C6—C7	1.3900	C10—C11	1.494 (4)
C6—H6	0.9300	C11—H11A	0.9600
C7—N1	1.382 (3)	C11—H11B	0.9600
S1—C1	1.728 (3)	C11—H11C	0.9600
S2—C1	1.654 (4)		
			
C3—C2—C7	120.0	N1—C1—S1	110.0 (2)
C3—C2—S1	129.62 (11)	S2—C1—S1	122.7 (2)
C7—C2—S1	110.38 (11)	N1—C8—C9	112.4 (3)
C4—C3—C2	120.0	N1—C8—H8A	109.1
C4—C3—H3	120.0	C9—C8—H8A	109.1
C2—C3—H3	120.0	N1—C8—H8B	109.1
C3—C4—C5	120.0	C9—C8—H8B	109.1
C3—C4—H4	120.0	H8A—C8—H8B	107.9
C5—C4—H4	120.0	C10—C9—C8	113.4 (3)
C4—C5—C6	120.0	C10—C9—H9A	108.9
C4—C5—H5	120.0	C8—C9—H9A	108.9
C6—C5—H5	120.0	C10—C9—H9B	108.9
C7—C6—C5	120.0	C8—C9—H9B	108.9
C7—C6—H6	120.0	H9A—C9—H9B	107.7
C5—C6—H6	120.0	O1—C10—C9	121.6 (2)
N1—C7—C6	127.51 (16)	O1—C10—C11	122.1 (3)
N1—C7—C2	112.49 (16)	C9—C10—C11	116.3 (3)
C6—C7—C2	120.0	C10—C11—H11A	109.5
C2—S1—C1	92.25 (14)	C10—C11—H11B	109.5
C1—N1—C7	114.8 (2)	H11A—C11—H11B	109.5
C1—N1—C8	121.3 (3)	C10—C11—H11C	109.5
C7—N1—C8	123.8 (3)	H11A—C11—H11C	109.5
N1—C1—S2	127.3 (3)	H11B—C11—H11C	109.5
			
C7—C2—C3—C4	0.0	C2—C7—N1—C1	1.5 (3)
S1—C2—C3—C4	−178.85 (17)	C6—C7—N1—C8	0.9 (4)
C2—C3—C4—C5	0.0	C2—C7—N1—C8	−179.6 (2)
C3—C4—C5—C6	0.0	C7—N1—C1—S2	−179.4 (2)
C4—C5—C6—C7	0.0	C8—N1—C1—S2	1.7 (5)
C5—C6—C7—N1	179.5 (2)	C7—N1—C1—S1	−1.8 (3)
C5—C6—C7—C2	0.0	C8—N1—C1—S1	179.3 (2)
C3—C2—C7—N1	−179.6 (2)	C2—S1—C1—N1	1.2 (2)
S1—C2—C7—N1	−0.53 (18)	C2—S1—C1—S2	178.9 (2)
C3—C2—C7—C6	0.0	C1—N1—C8—C9	85.9 (4)
S1—C2—C7—C6	179.05 (14)	C7—N1—C8—C9	−93.0 (3)
C3—C2—S1—C1	178.56 (17)	N1—C8—C9—C10	−175.5 (3)
C7—C2—S1—C1	−0.38 (15)	C8—C9—C10—O1	3.4 (5)
C6—C7—N1—C1	−178.0 (2)	C8—C9—C10—C11	−177.1 (3)

### Hydrogen-bond geometry (Å, °)

**Table:**

*D*—H···*A*	*D*—H	H···*A*	*D*···*A*	*D*—H···*A*
C11—H11B···O1^i^	0.96	2.60	3.541 (5)	167

Symmetry codes: (i) *x*+1, *y*, *z*.
